# Informed consent in randomised controlled trials: further development and evaluation of the participatory and informed consent (PIC) measure

**DOI:** 10.1186/s13063-023-07296-y

**Published:** 2023-05-02

**Authors:** Julia Wade, Elka Humphrys, Alba X Realpe, Daisy M Gaunt, Jenni Burt, on behalf of the OPTiMISE study group and the QuinteT research group, James P. Sheppard, Mark Lown, Eleanor Temple, Rebecca Lowe, Rosalyn Fraser, Julie Allen, Gary A Ford, Carl Heneghan, F. D. Richard Hobbs, Sue Jowett, Shahela Kodabuckus, Paul Little, Jonathan Mant, Jill Mollison, Rupert A. Payne, Marney Williams, Ly-Mee Yu, Richard J. McManus, Carmel Conefrey, Jenny Donovan, Nicola Farrar, Marcus Jepson, Ava Lorenc, Nicola Mills, Sangeetha Paramasivan, Leila Rooshena

**Affiliations:** 1 Bristol Medical School, University of Bristol, 39 Whatley Road, Clifton, Bristol BS8 2PS UK; 2 THIS Institute (The Healthcare Improvement Studies Institute), University of Cambridge, Strangeways Research Laboratory, Cambridge, CB1 8RN UK; 3 NIHR Bristol Biomedical Research Centre, University Hospitals Bristol and Weston NHS Foundation Trust and University of Bristol, Bristol, UK

**Keywords:** Informed consent, Recruitment, Communication

## Abstract

**Background:**

Informed consent is an accepted ethical and legal prerequisite for trial participation, yet there is no standardised method of assessing patient understanding for informed consent. The participatory and informed consent (PIC) measure was developed for application to recruitment discussions to evaluate recruiter information provision and evidence of patient understanding. Preliminary evaluation of the PIC indicated the need to improve inter-rater and intra-rater reliability ratings and conduct further psychometric evaluation. This paper describes the assessment, revision and evaluation of the PIC within the context of OPTiMISE, a pragmatic primary care-based trial.

**Methods:**

This study used multiple methods across two phases. In phase one, one researcher applied the existing PIC measure to 18 audio-recorded recruitment discussions from the OPTiMISE study and made detailed observational notes about any uncertainties in application. Appointments were sampled to be maximally diverse for patient gender, study centre, recruiter and before and after an intervention to optimise information provision. Application uncertainties were reviewed by the study team, revisions made and a coding manual developed and agreed. In phase two, the coding manual was used to develop tailored guidelines for applying the PIC to appointments within the OPTiMISE trial. Two researchers then assessed 27 further appointments, purposively sampled as above, to evaluate inter-rater and intra-rater reliability, content validity and feasibility.

**Results:**

Application of the PIC to 18 audio-recorded OPTiMISE recruitment discussions resulted in harmonisation of the scales rating recruiter information provision and evidence of patient understanding, minor amendments to clarify wording and the development of detailed generic coding guidelines for applying the measure within any trial. Application of the revised measure using these guidelines to 27 further recruitment discussions showed good feasibility (time to complete), content validity (completion rate) and reliability (inter- and intra-rater) of the measure.

**Conclusion:**

The PIC provides a means to evaluate the content of information provided by recruiters, patient participation in recruitment discussions and, to some extent, evidence of patient understanding. Future work will use the measure to evaluate recruiter information provision and evidence of patient understanding both across and within trials.

**Supplementary Information:**

The online version contains supplementary material available at 10.1186/s13063-023-07296-y.

## Background

Informed consent is a legal and ethical imperative and a prerequisite for all trial recruitment, as enshrined in national and international guidelines [1–3]. Informed consent requires voluntariness, capacity, disclosure, understanding and decision [4]. The quality of informed consent is determined by what information is provided and how it is presented (disclosure) and by the extent of patient understanding that arises as a result (understanding) [5–7]. In the context of ensuring informed consent for clinical trial participation, much attention is paid to monitoring written disclosure, with scrutiny of patient information leaflets (PILs) by ethics committees and review boards [8, 9], despite evidence that PILs do not necessarily serve to facilitate informed decisions about participation [10, 11]. Less attention is paid to monitoring the content of spoken information or patient understanding as it emerges during recruitment and informed consent discussions, the content of which is largely left to the discretion of individual recruiters [12].

A systematic review and meta-analysis of patient understanding for informed consent in clinical trials found that the proportion of patients who understood key components for informed consent varied from 52.1 to 75.8%; the review concluded that recruiters could do more to support better understanding [13]. Systematic reviews of interventions to improve research participants’ understanding in informed consent indicate that extended discussion may be effective [14, 15]. Existing measures to evaluate the information provision process during recruitment to research assess either the provision of information by recruiters [12] or patient recall of information [16–20]. Current methods of monitoring patient understanding for informed consent in research rely predominantly on patient self-report questionnaires [16, 17] or interviews conducted after the recruitment and informed consent discussion [19, 20]. There has been little attempt to evaluate the provision of information by recruiters and subsequent participant understanding. Yet empirical evidence shows that recruiters’ presentation of information to potential trial participants can have considerable impact on participant understanding of key concepts such as equipoise or randomisation [21, 22]. A tool which evaluates how recruiters present information to potential participants and how this impacts on evidence of participant understanding could provide valuable feedback in training recruiters in information provision, not just within trials research but research more generally.

The participatory and informed consent (PIC) measure was developed to assess the quality of information provision as evidenced in trial recruitment appointments, including evaluation of patient contributions during the interaction. Its aim is to evaluate the content and clarity of information provided by recruiters and how this impacts participant understanding, as evidenced in the discussion. Initial evaluation of the developmental version of the PIC showed promising validity and reliability when applied to six appointments from three secondary care randomised trials [23]. The measure was designed for application to the audio recordings or transcript of trial recruitment and informed consent discussions (i.e. any consultation during which trial participation is discussed between a healthcare professional and a potential participant). It required rating of recruiter information provision and evidence of participant understanding on a 4-point scale across 22 parameters (23 parameters for 3 group trials) and is described in detail elsewhere [23]. Previous work highlighted the need to develop a detailed coding manual with the objective of making application of the measure transparent so that unfamiliar users can apply it with consistency.

This paper describes the further development of the PIC measure, aiming to produce a refined measure suitable for application to any trial with high validity and reliability. To facilitate this development, we drew on a sample of recruitment appointments audio recorded within the OPTiMISE study, a pragmatic primary care-based randomised controlled trial (RCT), evaluating the safety of reducing blood pressure medication in patients with stable blood pressure aged 80 or over [24].

## Methods

This study used multiple methods across two phases. Phase one involved the application of the existing PIC measure by a researcher with no previous experience of the measure (EH) to a purposive sample of 18 audio-recorded recruitment appointments, the completion of an observation log on issues arising, and iterative consensus meetings within the team, with the aim of identifying amendments to the measure and developing detailed guidelines for its application (a coding manual). Phase two involved the evaluation of the validity and reliability of the amended PIC when applied to a further 27 purposively sampled recruitment appointments by the same researcher involved in phase one (EH) and a second researcher with no previous experience of applying the measure (AR).

### Materials

Recruitment appointments were sampled from those audio recorded during recruitment to the OPTiMISE RCT; all involved discussion between a GP and an eligible patient [24]. Eligible patients were sent a letter of invitation and those responding were invited to attend a screening appointment with their GP. As part of the recruitment discussion that took place during that screening appointment, all patients were shown a 2-min online OPTiMISE study video (see Additional file 1 for video script). The video described the purpose of the study as being to examine whether it was possible to safely reduce the number of drugs prescribed to people over the age of 80, who have blood pressure within normal range and who are taking two or more medications to reduce blood pressure. It was explained that too many blood pressure medications may be associated with increased risk of falls. The two arms (referred to as ‘groups’) were described as the ‘intervention group’ (one blood pressure medication removed) and the ‘control group’ (continue with current medication as usual). The video also explained randomisation in the following terms: participants would be randomly allocated to one of the above two groups. Participants would not be able to decide which group they were in and neither would their doctor or any of the research team (Additional file 1). Understanding of information provided in the video and patient information leaflet was explored and patients had the opportunity to ask questions about the study before deciding about participation. This discussion was audio recorded, with consent, and transcribed verbatim.

### Phase one: formative assessment of the PIC measure and development of a coding manual

The aim in phase one was to conduct a formative assessment of the PIC and identify content required for inclusion in a detailed coding manual to enable application of the PIC by a researcher with no previous knowledge of the measure. The DevPIC, as previously published, required the rater to evaluate content and clarity of recruiter information provision and patient understanding on two separate 4-point scales across 22 parameters (25 parameters in 3 arm studies) [23]. It also required global judgements, from the observed interaction, as to whether (i) the recruiter and (ii) the potential participant appeared to be in equipoise, (iii) whether the latter accepted random allocation as an acceptable way of determining treatment and (iv) whether they appeared to be adequately informed to be able to reach a decision about participation.

The PIC was applied by EH, a social scientist and member of the OPTiMISE study team with no previous experience of using the PIC, to evaluate 18 audio-recorded OPTiMISE recruitment discussions between GPs and eligible patients [24]. The sample was chosen using principles of maximum variation, to include those conducted by a range of GP recruiters, from a range of centres recruiting to OPTiMISE, including longer and shorter discussions, a mix of male and female patients and appointments sampled before and after a checklist intervention was disseminated to GP recruiters with the aim of optimising information provision. In doing so, a log, in the form of a free text, reflexive journal, was kept of how the measure was applied and all questions that arose in application and interpretation of the PIC parameters or scales, so that these issues could inform both revisions to the PIC measure and the development of any guidelines on how to apply the measure. Consensus meetings included two remote meetings between EH and JW to discuss these questions and potential amendments to the PIC, followed by a face-to-face meeting involving EH, JW, AR and JB in which all questions and potential amendments were discussed and a solution agreed. Solutions took the form of amendments to the PIC or clarifications to processes for applying the PIC as described within the coding manual.

### Phase two: evaluation of feasibility, validity and reliability

The revised PIC was then applied, using the coding guidelines developed above, independently by two raters (EH and AR, who had no previous experience of applying the measure) to a sample of 27 audio-recorded and transcribed recruitment discussions, again sampled to include a maximally diverse sample as described above and including appointments conducted before and after a checklist intervention designed to optimise GP information provision was disseminated to recruiters. As part of the OPTiMISE social science team, EH was not blinded to the pre/post intervention categorisation, but AR was. The inclusion of the OPTiMISE video embedded within recruitment discussions was accommodated by raters evaluating the content of the video independently first. The ‘live’ recruitment discussion was then rated for each recruitment discussion and could override the baseline evaluation of the content of the video. We evaluated the feasibility, validity and reliability of the measure in the following ways:

- *Feasibility*: the length of each recruitment discussion and the time taken to apply the measure to each discussion were recorded to evaluate feasibility in applying the measure.
- *Validity*: response rates and missing data were identified for individual items to evaluate their acceptability.
- *Reliability*: inter-rater reliability was assessed by comparing item responses made independently by the two raters. Each rater was required to rate each of 22 parameters twice (once rating recruiter information provision and once rating evidence of patient understanding as shown in patient talk) giving a total of 44 ratings per appointment. In determining reliability (and stability, see below), a discrepancy of 1 point or less was deemed acceptable on the grounds that this might represent the difference between presence or absence of information or between minimal and adequate information on the scale.
- *Stability*: stability (test–retest) reliability of the new measure was assessed by evaluating change in item responses when the measure was applied to a single recruitment discussion by the same researcher (AR), with an interval of at least 14 days between applications. Rating procedure was as described for inter-rater reliability.
- *Global judgements:* Raters were asked to judge four global parameters regarding evidence of recruiter and participant equipoise, participant acceptance of randomisation and participant understanding for IC. Response categories were ‘Yes’ (sufficient evidence), ‘No’ or ‘insufficient evidence’.

## Results

### Phase one: amendments to the PIC and development of coding manual

Characteristics of the 18 OPTiMISE recruitment appointments to which the PIC was applied are shown in Table 1. Following completion of the evaluation of these 18 appointments, the log of issues arising was reviewed by JW, EH, AR and JB to agree any modifications to the measure that were found to improve clarity of application and determine the content of a coding manual to provide guidelines to a new user on how to apply the measure. Issues and responses are described below.

**Table 1:** Characteristics of recruitment discussions evaluated in phase one

*n* = 18 recruitment discussions	
Patient gender	
F	13
M	5
Patients per study centre (*n* = 12 centres)	
(1 recruiter per study centre)	
A	3
B	1
C	2
D	1
E	1
F	0
G	3
H	0
I	4
J	1
K	1
L	1
Pre or post checklist intervention	
Pre	11
Post	7
Participation in trial	
Yes	17
No	1
Length of discussion (min)	
Mean	10:31
Range	5:33–16:33
Time taken to complete (min)	
Rater 1 mean	52
Range	30–120
Rater 2 mean	56
Range	15-130

### Differences between coding scales

Both the clarity of recruiter information provision and evidence of participant understanding were rated on 4-point scales in the published version of the DevPIC v2 (Additional file 2, [23]). However, descriptors differed between the two scales. Whilst the scale evaluating evidence of participant understanding gave the opportunity to rate for evidence of *misunderstanding*, the scale evaluating recruiter information provision did not include the option to rate for evidence of *misleading information*, and this was highlighted as an inconsistency and limitation. The measure was therefore modified to harmonise descriptors across the two scales, with these allowing for rating of misleading information provision and evidence of misunderstanding respectively (Additional file 2). The category ‘misleading information’ captured lack of clarity. Guidance for rating recruiter information provision included the instruction that if incorrect information was given and not clarified by the end of the discussion, then the parameter should be rated as 0. It was argued that this harmonisation of descriptors across the two scales would make the measure easier to apply.

### Review of wording

Wording of all parameters was reviewed to ensure maximum clarity to raters and the following changes were agreed:

i) The word ‘trial’ was replaced by the word ‘study’ in all parameters. Although less specific, the term ‘study’ has been found to be less easily misinterpreted by potential participants [22]. Given that the PIC was conceptualised as a tool to encourage a more participant-centred approach to information provision, the term ‘study’ was adopted consistently throughout the measure, with the explicit intention of encouraging recruiters to use the term.
ii) Two parameters (Additional file 2, Sect. 2: 23, 25) were reworded to clarify meaning. One required evaluation of information/understanding regarding conflict of interests and this was made more explicit. The other required evaluation of information/understanding about what happened if anything went wrong. Both parameters were reworded in line with text conventionally used in PILs [9] as it was argued that this was an accepted plain English version.

### Development of generic guidelines

Existing DevPIC guidelines explained that the measure was designed to be applied to the audio recording or transcript of recruitment discussion, that ratings should reflect all talk within a recorded discussion relevant to any one parameter, but that any segment of talk could be rated against more than one parameter and gave explanations to guide rating of each parameter [23]. Application of the PIC to the OPTiMISE study data highlighted the need to create more explicit guidelines to guide raters, in particular guidelines on what contributions should be rated minimal and which should be rated adequate. Data from the 18 OPTiMISE appointments rated minimal and adequate were reviewed by JW to identify the key content for each parameter that distinguished ‘adequate’ from ‘minimal’ and summarised as a full set of generic guidelines for discussion and final agreement with EH, JB and AR. The final set of agreed guidelines is shown in Additional file 3 (presented against a white background).

Having agreed these generic guidelines, the template shown in Additional file 3 was applied by EH and JB to draw up tailored guidelines for how these parameters should be evaluated within the specific context of the OPTiMISE study. Tailored guidelines were developed by EH and JB by reviewing the content of key study documents, including the PIL, the online video and the study protocol. Tailored guidelines were then agreed with the broader OPTiMISE trial team and with JW and AR. The final set of agreed guidelines for OPTiMISE is shown in Additional file 3 (presented against a blue background).

### Phase two: evaluation of feasibility, reliability and stability

The revised PICv3 (Additional file 2) was applied to a further 27 audio-recorded and transcribed recruitment discussions sampled from the OPTiMISE study, using the guidelines customised to OPTiMISE shown in Additional file 3. The characteristics of these recruitment discussions are shown in Table 2.

**Table 2:** Characteristics of recruitment discussions evaluated in phase two

*n* = 27 recruitment discussions	
Patient gender	
F	11
M	16
Patients per study centre (*n* = 12 centres)	
(1 recruiter per study centre)	
A	0
B	3
C	5
D	5
E	1
F	1
G	3
H	2
I	5
J	0
K	1
L	1
Pre or post checklist intervention	
Pre	19
Post	8
Participation in trial	
Yes	26
No	1
Length of discussion (min)	
Mean	12
Range	7–24
Time taken to complete (min)	
Rater 1 mean	42
Range	15–70
Rater 2 mean	52
Range	30–120

### Feasibility

Time taken to complete PIC is shown in Table 2. The mean length of the audio-recorded discussions was 12 (range 7–24) min. For these, the mean completion time was 42 (range 15–70) min and 52 (range 30–120) min for raters 1 and 2, respectively. This was a reduction in the mean time taken to complete compared with the previously recorded mean rating time of 56 min applying the DevPIC to appointments [23]. However, the mean length of appointments to which the DevPIC was previously applied (22, range 14–66 min) was nearly twice the mean length of appointments in the current study.

### Validity

Analysis of missing data for individual items showed no missing data in application of the measure by either rater, indicating good acceptability of included items.

### Reliability

Inter-rater reliability is shown in Table 3. Inter-rater agreement showed a discrepancy of 1 point or less in 94.44% (561/594) of ratings of recruiter information provision and 92.59% (550/594) of participant understanding. This was an increase upon levels of agreement of 90.58% (125/138) and 90.58% (125/138) respectively when previously evaluated [23].

**Table 3:** Inter and test–retest comparisons

	**Total *N* comparisons = 594**	***N* comparisons showing no discrepancy**	***N* comparisons showing 1-point discrepancy**	***N* comparisons showing 2-point discrepancy**	***N* comparisons showing 3-point discrepancy**
Inter-rater reliability	Recruiter information	453 (76%)	108 (18%)	30 (5%)	3 (< 1%)
	Patient understanding	365 (61%)	185 (31%)	42 (7%)	2 (< 2%)
Test–retest reliability	Recruiter information	487 (82%)	77 (13%)	26 (5%)	4 (< 1%)
	Patient understanding	389 (65%)	169 (28%)	33 (6%)	3 (< 1%)

### Stability of the measure

Rates of test–retest (intra-rater agreement) are shown in Table 3. Test-–retest agreement showed a discrepancy of 1 point or less in 94.95% (564/594) of ratings of recruiter information provision and 93.94% (558/594) of ratings of evidence of patient understanding. This compared to levels of test–retest agreement of 99.28% (137/138) and 99.28% (137/138) respectively when previously assessed [23].

### Analysis of global judgements

Ratings of global judgements are shown in Table 4. Levels of agreement were considerably lower for these ratings than for the rest of the measure. Greatest levels of inter-rater agreement were shown for ratings of participant acceptance of randomisation 63% (17/27) and participant equipoise 59% (16/27). Levels of agreement for evidence of sufficient participant understanding for IC and evidence of recruiter equipoise were 41% (11/27) and 37% (10/27), respectively. Qualitative comments raised concerns about overlap of the first global judgement with item 5 in Sect. 2 of the measure, evaluating recruiter communication of equipoise. Given these findings, it was suggested that global judgement 1 (evidence of recruiter equipoise) should be removed from the global judgements. The remaining three global judgements require judgements about evidence of three aspects of patient beliefs and understanding: (1) of patient equipoise, (2) of the patient finding randomisation an acceptable way to determine their treatment and (3) of the patient being sufficiently informed by the end of the consultation to make an informed decision about participation. These parameters are not assessed elsewhere in the measure yet are fundamental in evaluating patient preparedness to give informed consent so were retained. Lower levels of agreement on these ratings may reflect that these parameters are more difficult to operationalise or may reflect a need to clarify rater guidelines for these ratings.

**Table 4:** Comparison of ratings of global judgements

	**Recruiter equipoise**	**Participant equipoise**	**Participant accepts randomisation**	**Participant informed**
*N* of consultations	27	27	27	27
*N* of consultations showing no discrepancies in rating	10 (37%)	16 (59%)	17 (63%)	11 (41%)
*N* of consultations showing a discrepancy in rating	17 (63%)	11 (41%)	10 (37%)	16 (59%)

## Discussion

This study describes the further development and evaluation of the measure of participatory and informed consent (PIC), a tool developed to enable assessment of recruiter information provision and evidence of patient understanding during trial recruitment discussions. The original scale was applied by a researcher with no previous experience of the measure and modified in three ways: homogenisation of the rating scales across rating of recruiter information provision and evidence of patient understanding, clarification of wording describing dimensions assessed and development of a detailed generic coding manual to guide application of the measure to any trial. This manual was then used to create trial specific guidelines for application of the measure within the primary care based OPTiMISE trial. Evaluation of the measure as applied to recruitment discussions by the same researcher and one other researcher, also with no previous experience of the measure, within OPTiMISE showed excellent levels of agreement for inter-rater reliability and test–retest reliability for users without prior knowledge of the tool, with no adverse impact on time taken to complete. These guidelines for application and high levels of inter-rater and test–retest reliability will enable the measure to be applied to compare and contrast recruiter information provision and evidence of patient understanding within trials, for example before and after interventions to optimise recruiter information provision [25] and across trials to compare and optimise practice.

Evidence suggests that optimising recruitment and informed consent discussions can bring improved patient understanding for informed consent during trial recruitment [14, 15]. Based on this evidence, the PIC was conceived as a tool to evaluate information that recruiters provided whilst simultaneously evaluating what evidence emerged of patient understanding of this information. This study showed excellent inter-rater and test–retest agreement across ratings of both recruiter information provision and evidence of patient understanding, although values were greater than 90% for both. Information provision for informed consent will always be a process rather than a single event; the degree to which evidence of patient understanding as measured in the PIC corresponds to patient understanding as evidenced in patient self-report questionnaires has yet to be evaluated. We acknowledge the methodological challenges inherent in attempting to evaluate patient understanding based on patient contributions made during a recruitment and informed consent discussion. Minimal patient contributions such as continuers (*uhuh, mm, ok, right*) may be interpreted as claims to understanding rather than as demonstration of understanding [26]. Patient self-completion questionnaires for evaluating understanding for informed consent may provide a better profile of true patient understanding [16–18]. However, these methods require completion by patients and therefore are an added burden in addition to trial questionnaires. Our focus on evidence of patient understanding as it emerged in the recruitment discussion was predicated on this being the only evidence available routinely to recruiters in making judgements as to whether a person was sufficiently informed or not. It may be that the PIC whilst aiming to capture evidence of *patient understanding* can, in effect, only claim to capture evidence of *patient participation* in the interaction. We argue this is still a goal worth pursuing. An extensive pedagogical literature documents how people acquire knowledge and understanding most effectively when they engage actively with the material to be understood [27–29]. There is evidence that listening to a monologue delivered by an expert is less conducive to acquiring understanding than encouraging the learner to generate their own questions [30, 31]. Previous work has highlighted variation in the extent to which patients take an active role in recruitment discussions and identified recruiter behaviours which may impact on how actively participants engage in the discussion [32]. It may be that in future, the PIC tool can be used to support training interventions to move recruiters away from delivering a monologue and towards a more interactive format that will promote better understanding for patients.

Given the promising reliability and stability demonstrated by this work, the PIC measure can now be used to evaluate and inform recruiter practice. The measure provides a total score for recruiter information provision and total score for evidence of participant understanding and gives total scores for each of these across the domains of scene setting, study treatments and study procedures for cross comparison between trial recruitment consultations or between recruiters. We would, however, argue against setting a threshold value, above which either recruiter information provision or evidence of participant understanding is deemed acceptable, so acknowledge that the measure is not conceived as being a summative assessment but as a formative one. It perhaps most usefully functions to identify gaps in routine information provision or evidence of understanding and make explicit the relationship between scores for recruiter information provision and scores for evidence of participant understanding across its 22 parameters. Other omissions, such as failing to outline the benefits or advantages of a treatment/management option, are common across trials and may be the result of poor recruiter confidence in conveying these concepts clearly [25]. By highlighting and addressing the information topics that are commonly omitted, it will be possible to identify both cross trial and trial specific areas that can be addressed via recruiter training. Further research is required to evaluate the sensitivity of the measure in evaluating training effects on both recruiter information provision and evidence of patient understanding. Future work should also examine whether the measure can be shortened. Items 1–8 contained in Sect. 2, part i (scene setting) may function as a brief assessment of the quality of information provided and a guide as to whether entire application of the measure is required or not. The relationship between scores for recruiter information provision and evidence of participant understanding should also be explored. It is reasonable to hypothesise that where participants contribute little to the discussion, there will be a greater discrepancy between recruiter and participant score and where there is more engagement by participants, the participant score will fall closer to the recruiter score, but this will require further evaluation. Finally, future work should also investigate how the measure can be applied to research involving human subjects other than trials research. Only three parameters within the PIC v3 evaluated information provision which is only relevant within a trials research context: 5. Clinical equipoise; 7. Reason for randomisation and 8. Process of randomisation; the remaining parameters remain valid for consenting to non-trials research, so the measure may function equally in this context with these 3 parameters omitted.

This study had limitations. We found lower levels of test–retest agreement than the previous evaluation of the DevPIC: a discrepancy of 1 point or less in 94.95% (564/594) of ratings of recruiter information provision and 93.94% (558/594) of ratings of evidence of patient understanding, compared to levels of test–retest agreement of 99.28% (137/138) and 99.28% (137/138) respectively on previous assessment [23]. However, the absolute percentage level agreement remained greater than 90% and reflected test–retest agreement evaluated over 27 recruitment discussions (compared to only six in the previous study) so can be taken as a more representative figure. The study design did not include evaluation of validity by comparing the performance of the PIC to the previously used comparator tool, the P-QIC [12]. Validation studies of findings will need to be carried out elsewhere. However, previous comparison had shown good correlation of P-QIC and DevPIC scores for recruiter information provision [23] and low levels of missing data on this evaluation were encouraging for validity of the measure. Future evaluation might include comparison of PIC scores with measures of understanding for informed consent using patient self-report data. Levels of agreement on the global judgements (see Additional file 1 p5) showed considerably lower levels of agreement yet three of these judgements evaluate evidence of patient views or understanding that are central to understanding for informed consent to trial participation and for this reason, it was not felt that these could be removed from the measure. Future research will need to explore whether clearer guidelines on completion of these ratings can bring greater levels of inter-rater and test–retest agreement and how ratings on these parameters compare to ratings in Sect. 2 of the measure. We also note the relatively low level of inter-rater agreement (41%) on the global judgement: *Do you believe that the patient is sufficiently informed by the end of the consultation to make an informed decision?* This finding is problematic if the PIC is to make any claims to measure evidence of patient understanding.

## Conclusions

The PIC is a measure of recruiter information provision and patient participation designed to be applied to an audio recording or a transcription of a recruiter discussion for a trial. It is the only measure of its kind to attempt to measure patient participation and evidence of understanding as demonstrated within the recruitment discussion. In the latest version presented here, it includes a coding manual to enable application to any novel trial context. Where this coding manual is applied to create trial specific guidelines in scoring, excellent levels of inter-rater and test–retest reliability can be achieved. Future evaluation of the measure needs to include assessment of its ability to distinguish between a range of practice in terms of recruiter information provision both with trials and across trials, shortening of the measure to increase feasibility of application and comparison of how well the measure of evidence of patient understanding compares to evaluation of this via self-report questionnaire.

## Supplementary Information

**Additional file 1. **OPTiMISE video script.

**Additional file 2. **DevPIC version2.

**Additional file 3. **PIC version3.

**Additional file 4. **PIC version 3 instructions for use.

## Data Availability

Owing to the conditions of the ethical approval for the project, the raw data analysed here (audio recordings of consent consultations) are not available for deposit. This is due to the sensitive nature of the data and concerns that it would be difficult to completely de-identify participants. Any requests for access to or use of the data should be made to the corresponding author. Access to fully anonymised data for suitable purposes may be granted to bona fide researchers under a data sharing agreement, subject to approval from relevant ethics committee/s.

## Abbreviations

RCT: Randomised controlled trial
PIC: Participatory informed consent
PIL: Patient information leaflet
P-QIC: Process and quality of informed consent

## References

[1] World Medical Association Declaration of Helsinki. Ethical principles for medical research involving human subjects. Adopted by the 18th World Medical Assembly, Helsinki, Finland, June 1964 and amended 2013. Available at http://www.wma.net/en/20activities/10ethics/10helsinki/. Accessed 7 Jan 2016.

[2] Council for International Organizations of Medical Sciences (CIOMS). International Ethical Guidelines for Biomedical Research Involving Human Subjects. 2002, Geneva, Switzerland. Available at http://www.cioms.ch/publications/layout_guide2002.pdf. Accessed 14983848

[3] US Department of Health and Human Services. Code of Federal Regulations, 21 Part 50 Protection of Human Subjects and 45 part 46 Protection of Human Subjects, Federal Register 1991 June 18; 56: 28012. Available at https://www.accessdata.fda.gov/scripts/cdrh/cfdocs/cfcfr/CFRSearch.cfm?CFRPart=50. Accessed

[4] Beauchamp TL, Childress JF (1994). Principles of biomedical ethics.

[5] Sreenivasan G (2003). Does informed consent to research require comprehension?. Lancet.

[6] Lad PM, Dahl R (2014). Audit of the informed consent process as a part of a clinical research quality assurance program. Sci Eng Ethics.

[7] Richardson V (2013). Patient comprehension of informed consent. J Perioper Pract..

[8] US Food and Drug Administration, Department of Health and Human Services. Draft guidance: informed consent information sheet. Guidance for IRBs, clinical investigators, and sponsors. Silver Spring, MD: US Food and Drug Administration, Department of Health and Human Services; 2014. https://www.fda.gov/regulatory-information/search-fda-guidance-documents/guide-informed-consent#content. Accessed 04/02/20

[9] National Health Service Health Research Authority. Consent and Participant Information Sheet Preparation Guidance https://www.hra.nhs.uk/planning-and-improving-research/best-practice/informing-participants-and-seeking-consent/ Accessed 04/02/2020

[10] Do informed consent documents for cancer trials do what they should? A study of manifest and latent functions Sociology of Health and Illness. 2012 | journal-article: 10.1111/j.1467-9566.2012.01469.x 10.1111/j.1467-9566.2012.01469.x 22443456

[11] Gillies K, Huan W, Skea Z, Brehaut J, Cotton S (2014). Patient information leaflets (PILs) for UK randomised controlled trials: a feasibility study exploring whether they contain information to support decision making about trial participation. Trials.

[12] Cohn EG, Jia H, Chapman Smith W, Erwin K, Larson EL (2011). Measuring the process and quality of informed consent for clinical research: development and testing. Oncol Nurs Forum.

[13] Tam NT, Huy NT, Thoa LTB, Long NP, Trang NTH, Hirayama K, Karbwang J (2015). Participants’ understanding of informed consent in clinical trials over three decades: systematic review and meta-analysis. Bulletin World Health Organisation.

[14] Nishimura A, Carey J, Erwin PJ (2013). Improving understanding in the research informed consent process: a systematic review of 54 interventions tested in randomized control trials. BMC Med Ethics.

[15] Flory J, Emanuel E (2004). Interventions to improve research participants’ understanding in informed consent for research: a systematic review. JAMA.

[16] Joffe S, Cook EF, Cleary PD, Clark JW, Weeks JC (2001). Quality of informed consent: a new measure among research subjects. J Natl Cancer Inst.

[17] Guarino P, Lamping DL, Elbourne D, Carpenter J, Peduzzi P (2006). A brief measure of perceived understanding of informed consent in a clinical trial was validated. J Clin Epidemiol.

[18] Hutchison C, Cowan C, Paul J (2007). Patient understanding of research: developing and testing of a new questionnaire. Eur J Cancer Care.

[19] Sugarman J, Lavori PW, Boeger M, Cain C, Edson R, Morrison V, Yeh SS (2005). Evaluating the quality of informed consent. Clin Trials.

[20] Kass NE, Taylor HA, Ali J, Hallez K, Chaisson L (2015). A pilot study of simple interventions to improve informed consent in clinical research: feasibility, approach, and results. Clin Trials.

[21] Rooshenas  L, Elliott D, Wade J, Jepson M, Paramasivan S, Strong S, Wilson C, Beard D, Blazeby JM, Birtle A, Halliday A, Rogers CA, Stein R, Donovan JL (2016). Conveying equipoise during recruitment for clinical trials: qualitative synthesis of clinicians’ practices across six randomised controlled trials. PloS Medicine.

[22] Jepson M, Elliott D, Conefrey C, Wade J, Rooshenas L, Wilson C, Beard D, Blazeby JM, Birtle A, Halliday A, Stein R, Donovan JL; CSAW study group; Chemorad study group; POUT study group; ACST-2 study group; OPTIMA prelim study group. An observational study showed that explaining randomization using gambling-related metaphors and computer-agency descriptions impeded randomized clinical trial recruitment. J Clin Epidemiol. 2018;99:75-83. 10.1016/j.jclinepi.2018.02.018. 10.1016/j.jclinepi.2018.02.018 PMC6015122 29505860

[23] Wade J, Elliott D, Avery KNL, Gaunt D, Young GJ, Barnes R, Paramasivan S, Campbell WB, Blazeby JM, Birtle AJ, Stein RC, Beard DJ, Halliday AW, Donovan JL; ProtecT study group; CLASS study group; Chemorad study group; POUT study group; OPTIMA prelim study group; CSAW study group and ACST-2 study group. Informed consent in randomised controlled trials: development and preliminary evaluation of a measure of Participatory and Informed Consent (PIC). Trials. 2017;18(1):327. 10.1186/s13063-017-2048-7. 10.1186/s13063-017-2048-7 PMC5513045 28716064

[24] Sheppard JP, Burt J, Lown M (2018). OPtimising Treatment for MIld Systolic hypertension in the Elderly (OPTiMISE): protocol for a randomised controlled non-inferiority trial. BMJ Open.

[25] Mills N, Gaunt D, Blazeby JM, Elliott D, Husbands S, Holding P (2018). Training health professionals to recruit into challenging randomized controlled trials increased confidence: the development of the QuinteT randomized controlled trial recruitment training intervention. J Clin Epidemiol.

[26] Sacks H (1992). Lectures on conversation.

[27] Ambrose SA, Bridges MW, DiPietro M, Lovell MC, Norman MK, Mayer RE (2010). How learning works: 7 research based principles for smart teaching.

[28] Prince M (2004). Does active learning work? A review of the research. J Eng Educ.

[29] Bonwell CC, Eison JJ, Active learning: creating excitement in the classroom (1991). ASH#-ERIC, Higher Education report No 1.

[30] Chi, M. T. H., Roy, M., & Hausmann, R. G. M. (2008). Observing tutorial dialogues collaboratively: insights about human tutoring effectiveness from vicarious learning. Cognitive Science, 33, 301– 341.or self‐generate questions 10.1080/03640210701863396 21635338

[31] King A (1992). Comparison of self-questioning, summarizing, and notetaking-review as strategies for learning from lectures. Am Educ Res J..

[32] Wade J, Donovan JL, Lane JA, Neal DE, Hamdy F (2009). It's not just what you say, it's also how you say it: opening the 'black box' of informed consent appointments in randomised controlled trials. Soc Sci Med.

