# Supplementary material for: Informed consent in randomised controlled trials: further development and evaluation of the participatory and informed consent (PIC) measure
Source: Trials. 2023 May 2;24:305. doi: 10.1186/s13063-023-07296-y (PMC10155434; doi:10.1186/s13063-023-07296-y)
Supplement: Supplementary file 1 — Additional file 1. OPTiMISE video script. [file 13063_2023_7296_MOESM1_ESM.docx]

**OPTiMISE video infographic script**

**Introduction**

Sustained high blood pressure can lead to heart attacks and stroke. It is one of the most common medical conditions in older people and many take two or more tablets to reduce it.

Recent scientific research suggests that large reductions in blood pressure, and too many drug prescriptions may be associated with an increased risk of falls and even death in older patients, particularly in those suffering from lots of medical conditions.

We are conducting a research study to examine whether it is possible to safely reduce the number of drugs prescribed to people over the age of 80 who have blood pressure in a normal range and are taking two or more medications.

**What the study involves**

We would like to recruit 540 people to the study.

Once they have agreed to take part, they will be randomly allocated to one of two groups.

A ‘control’ group in which participants will continue with their current medication,

Or,

An ‘intervention’ group, where participants will have one blood pressure lowering drug removed, under the instruction of their doctor.

Participants will not be able to decide which group they would like to be in, neither can their doctor or any of the research team.

The trial will last for 12 weeks and participants will need to attend their GP surgery on a minimum of 3 occasions during this period.

At each visit, they will have their blood pressure measured and be asked to complete questionnaires about themselves.

**Summary**

We hope the OPTiMISE trial will improve the care of older people and the quality of life they enjoy.

To find out more, please visit us online or contact the research team directly.
