# Supplementary material for: Informed consent in randomised controlled trials: further development and evaluation of the participatory and informed consent (PIC) measure
Source: Trials. 2023 May 2;24:305. doi: 10.1186/s13063-023-07296-y (PMC10155434; doi:10.1186/s13063-023-07296-y)
Supplement: Supplementary file 2 — Additional file 2. DevPIC version2. [file 13063_2023_7296_MOESM2_ESM.docx]

**Appendix B** **DevPICv2**

**Participatory Informed Consent for trial recruitment**

**Part 1: Descriptive information about the consultation**

| Rater ID |  | | Date of rating | |  | |
| --- | --- | --- | --- | --- | --- | --- |
| Total time taken to rate | Start time (h:m) | | Finish time (h:m) | | Total time (h:m) | |
| Consultation ID |  | | Trial ID | |  | |
| Recruiter ID |  | | Recruiter’s Profession | |  | |
| Length of consultation (h:m) |  | | People present  R1 = recruiter  R2 = second recruiter  P1 = patient  P2= friend/family | |  | |
| Trial treatment arms | 1. | | | | | |
|  | 2. | | | | | |
|  | 3. | | | | | |
| Decision outcome in terms of participation | e.g. Unknown / Randomised & accepted allocation / Randomised & took time to consider / Randomised & rejected allocation / Refused randomisation & chose treatment / Undecided | | | | | |
|  | Scene setting | Study treatments | | Study procedures | |  |
| Score R info provision | Section 2 i a  /24 | Section 2 ii a  /18 /27 | | Section 2 iii a  /24 | | Total  /60 /69 |
| Score P understanding | Section 2 i a  /24 | Section 2 ii a  /18 /27 | | Section 2 iii a  /24 | | Total  /60 /69 |
| Comments e.g. analysis process, nature of consultation, quality of audio recording | | | | | | |

**Section 2: R information provision and evidence of P understanding**

**i) Scene setting:** consultation purpose, relevant history/diagnosis, management options, equipoise, reason for trial/randomisation

| **Topic** | 1. **R information provision is:** | 1. **P contributions suggest:**   **(circle most appropriate box)** | |
| --- | --- | --- | --- |
| 1. Purpose of consultation | 0 1 2 3 Absent Mostly unclear Mostly clear Very clear | Evidence of misunderstanding | 0 |
|  |  | No evidence available | 1 |
|  |  | Minimal evidence of understanding | 2 |
|  |  | Adequate evidence of understanding | 3 |
| 2. Relevant history: diagnosis and management to date | 0 1 2 3 Absent Mostly unclear Mostly clear Very clear | Evidence of misunderstanding | 0 |
|  |  | No evidence available | 1 |
|  |  | Minimal evidence of understanding | 2 |
|  |  | Adequate evidence of understanding | 3 |
| 3. Currently available management options within standard care | 0 1 2 3 Absent Mostly unclear Mostly clear Very clear | Evidence of misunderstanding | 0 |
|  |  | No evidence available | 1 |
|  |  | Minimal evidence of understanding | 2 |
|  |  | Adequate evidence of understanding | 3 |
| 4. Management options evaluated within trial | 0 1 2 3 Absent Mostly unclear Mostly clear Very clear | Evidence of misunderstanding | 0 |
|  |  | No evidence available | 1 |
|  |  | Minimal evidence of understanding | 2 |
|  |  | Adequate evidence of understanding | 3 |
| 5.Clinical equipoise (no evidence to suggest any treatment is better than other(s) ) | 0 1 2 3 Absent Mostly unclear Mostly clear Very clear | Evidence of misunderstanding | 0 |
|  |  | No evidence available | 1 |
|  |  | Minimal evidence of understanding | 2 |
|  |  | Adequate evidence of understanding | 3 |
| 6. Trial purpose or question (collect evidence as to whether one treatment is better than other(s)) | 0 1 2 3 Absent Mostly unclear Mostly clear Very clear | Evidence of misunderstanding | 0 |
|  |  | No evidence available | 1 |
|  |  | Minimal evidence of understanding | 2 |
|  |  | Adequate evidence of understanding | 3 |
| 7. Reason for randomisation (create two groups the same except for which treatment each group gets) | 0 1 2 3 Absent Mostly unclear Mostly clear Very clear | Evidence of misunderstanding | 0 |
|  |  | No evidence available | 1 |
|  |  | Minimal evidence of understanding | 2 |
|  |  | Adequate evidence of understanding | 3 |
| 8. Process of randomisation (must refer to chance allocation) | 0 1 2 3 Absent Mostly unclear Mostly clear Very clear | Evidence of misunderstanding | 0 |
|  |  | No evidence available | 1 |
|  |  | Minimal evidence of understanding | 2 |
|  |  | Adequate evidence of understanding | 3 |
| Total score | /24 | /24 | |
| **Comments arising during rating P2 i** | | | |

**ii) Trial treatments**

| **Topic** | 1. **R information provision is:** | 1. **P interaction suggests (tick box)** | |
| --- | --- | --- | --- |
| 9. Trial arm 1 processes | 0 1 2 3 Absent Mostly unclear Mostly clear Very clear | Evidence of misunderstanding | 0 |
|  |  | No evidence available | 1 |
|  |  | Minimal evidence of understanding | 2 |
|  |  | Adequate evidence of understanding | 3 |
| 10. Trial arm 1 costs or disadvantages | 0 1 2 3 Absent Mostly unclear Mostly clear Very clear | Evidence of misunderstanding | 0 |
|  |  | No evidence available | 1 |
|  |  | Minimal evidence of understanding | 2 |
|  |  | Adequate evidence of understanding | 3 |
| 11. Trial arm 1 benefits or advantages | 0 1 2 3 Absent Mostly unclear Mostly clear Very clear | Evidence of misunderstanding | 0 |
|  |  | No evidence available | 1 |
|  |  | Minimal evidence of understanding | 2 |
|  |  | Adequate evidence of understanding | 3 |
| 12. Trial arm 2 processes | 0 1 2 3 Absent Mostly unclear Mostly clear Very clear | Evidence of misunderstanding | 0 |
|  |  | No evidence available | 1 |
|  |  | Minimal evidence of understanding | 2 |
|  |  | Adequate evidence of understanding | 3 |
| 13. Trial arm 2 costs or disadvantages. | 0 1 2 3 Absent Mostly unclear Mostly clear Very clear | Evidence of misunderstanding | 0 |
|  |  | No evidence available | 1 |
|  |  | Minimal evidence of understanding | 2 |
|  |  | Adequate evidence of understanding | 3 |
| 14. Trial arm 2 benefits or advantages | 0 1 2 3 Absent Mostly unclear Mostly clear Very clear | Evidence of misunderstanding | 0 |
|  |  | No evidence available | 1 |
|  |  | Minimal evidence of understanding | 2 |
|  |  | Adequate evidence of understanding | 3 |
| 15. Trial arm 3 processes | 0 1 2 3 Absent Mostly unclear Mostly clear Very clear | Evidence of misunderstanding | 0 |
|  |  | No evidence available | 1 |
|  |  | Minimal evidence of understanding | 2 |
|  |  | Adequate evidence of understanding | 3 |
| 16. Trial arm 3 costs or disadvantages | 0 1 2 3 Absent Mostly unclear Mostly clear Very clear | Evidence of misunderstanding | 0 |
|  |  | No evidence available | 1 |
|  |  | Minimal evidence of understanding | 2 |
|  |  | Adequate evidence of understanding | 3 |

| 17. Trial arm 3 benefits or advantages | 0 1 2 3 Absent Mostly unclear Mostly clear Very clear | Evidence of misunderstanding | 0 |
| --- | --- | --- | --- |
|  |  | No evidence available | 1 |
|  |  | Minimal evidence of understanding | 2 |
|  |  | Evidence of understanding | 3 |
| Total score | /18 or /27 | /18 or /27 | |
| **Comments Part 2ii** | | | |

**Iii) Trial procedures**

| **Topic** | 1. **R information provision is:** | 1. **P interaction suggests:** | |
| --- | --- | --- | --- |
| 18. Advantages or benefits of trial participation | 0 1 2 3 Absent Mostly unclear Mostly clear Very clear | Evidence of misunderstanding | 0 |
|  |  | No evidence available | 1 |
|  |  | Minimal evidence of understanding | 2 |
|  |  | Adequate evidence of understanding | 3 |
| 19. Costs / risks of trial participation. | 0 1 2 3 Absent Mostly unclear Mostly clear Very clear | Evidence of misunderstanding | 0 |
|  |  | No evidence available | 1 |
|  |  | Minimal evidence of understanding | 2 |
|  |  | Adequate evidence of understanding | 3 |
| 20. Option to refuse participation. | 0 1 2 3 Absent Mostly unclear Mostly clear Very clear | Evidence of misunderstanding | 0 |
|  |  | No evidence available | 1 |
|  |  | Minimal evidence of understanding | 2 |
|  |  | Adequate evidence of understanding | 3 |
| 21. Option to withdraw from participation. | 0 1 2 3 Absent Mostly unclear Mostly clear Very clear | Evidence of misunderstanding | 0 |
|  |  | No evidence available | 1 |
|  |  | Minimal evidence of understanding | 2 |
|  |  | Adequate evidence of understanding | 3 |
| 22. Options for further consultation to support decision making | 0 1 2 3 Absent Mostly unclear Mostly clear Very clear | Evidence of misunderstanding | 0 |
|  |  | No evidence available | 1 |
|  |  | Minimal evidence of understanding | 2 |
|  |  | Adequate evidence of understanding | 3 |
| 23. Outlines benefits (financial or otherwise) to professional or organisation of P participation. | 0 1 2 3 Absent Mostly unclear Mostly clear Very clear | Evidence of misunderstanding | 0 |
|  |  | No evidence available | 1 |
|  |  | Minimal evidence of understanding | 2 |
|  |  | Adequate evidence of understanding | 3 |
| 24. Outlines measures to protect confidentiality of participant data | 0 1 2 3 Absent Mostly unclear Mostly clear Very clear | Evidence of misunderstanding | 0 |
|  |  | No evidence available | 1 |
|  |  | Minimal evidence of understanding | 2 |
|  |  | Adequate evidence of understanding | 3 |
| 25. Outlines measures for compensation in case of adverse events | 0 1 2 3 Absent Mostly unclear Mostly clear Very clear | Evidence of misunderstanding | 0 |
|  |  | No evidence available | 1 |
|  |  | Minimal evidence of understanding | 2 |
|  |  | Adequate evidence of understanding | 3 |
| Total score | /24 | /24 | |
| co**mments on Part 2iii** | | | |

**Section 3: Global Judgements**

**Please respond to the following questions as applied up to the point of decision making about whether to accept the option to randomize or the end of the appointment. Please circle your response and add any comments below.**

Does the recruiter consistently convey a position of equipoise and what evidence do you have to suggest this?

Yes Insufficient evidence No

Comments:

Do you believe the patient is in equipoise and what evidence do you have to suggest this?

Yes Insufficient evidence No

Comments:

Do you believe the patient accepts randomisation as a way to determine treatment and what evidence do you have to support this?

Yes Insufficient evidence No

Comments:

Do you believe that the patient is sufficiently informed by the end of the consultation to make an informed decision and what evidence to you have to support this?

Yes Insufficient evidence No

Comments:

**Section 4 Ethnographic commentary of what occurs within the consultation**.

There are no constraints on what can be included here. Feel free to add observations about **any elements of recruiter or patient contributions that stand out in this consultation** as regards **what** is discussed, **how** it is discussed and **how** it is understood.

Observations from this section will be used to develop the next version of the measure so please add comments on key issues that you feel need capturing and are not yet captured elsewhere. Shorthand notes are perfectly acceptable rather than written prose. It is intended that this section should be completed in around 10 minutes.
