# Supplementary material for: Informed consent in randomised controlled trials: further development and evaluation of the participatory and informed consent (PIC) measure
Source: Trials. 2023 May 2;24:305. doi: 10.1186/s13063-023-07296-y (PMC10155434; doi:10.1186/s13063-023-07296-y)
Supplement: Supplementary file 3 — Additional file 3. PIC version3. [file 13063_2023_7296_MOESM3_ESM.docx]

**Participatory and Informed Consent for trial recruitment**

**PICv3**

**Section 1: Descriptive information**

1. **Study and consultation**

| Study name |  | | | |
| --- | --- | --- | --- | --- |
| Rater ID |  | | Date of rating |  |
| Time taken to rate | Start time (h:m) | | Finish time (h:m) | Total time (h:m) |
| Consultation ID |  | | Study ID |  |
| Recruiter ID |  | | Recruiter’s Profession |  |
| Length of consultation (h:m) |  | | People present  R1 = recruiter  R2 = second recruiter  P1 = participant  P2 = friend/family |  |
| Study treatment arms | 1. |  | | |
|  | 2. |  | | |
|  | 3. |  | | |
| Decision outcome in terms of participation | e.g. Unknown / Randomised & accepted allocation / Randomised & took time to consider / Randomised & rejected allocation / Refused randomisation & chose treatment / Undecided | | | |

1. **Rating and comments**

| Score | Section 2 i:  Scene setting | | Section 2 ii:  Trial treatments | | Section 2 iii:  Trial procedures | | Total | |
| --- | --- | --- | --- | --- | --- | --- | --- | --- |
| Recruiter |  | /24 |  | /18 /27 |  | /24 |  | /66 /75 |
| Participant |  | /24 |  | /18 /27 |  | /24 |  | /66 /75 |
| Comments e.g. Analysis process, nature of consultation, quality of audio recording | | | | | | | | |

**Section 2: Recruiter information and participant understanding**

1. **Scene setting**

**Circle the most appropriate box for the information provided by the recruiter and the participant understanding for each item:**

| **Item** | **Recruiter information** | | | | **Participant understanding** | | | |
| --- | --- | --- | --- | --- | --- | --- | --- | --- |
|  | Mis-  Information^1^ | Absent | Minimal  information | Adequate  information | Mis-  understanding | Absent | Minimal understanding | Adequate understanding |
| 1. Purpose of consultation | 0 | 1 | 2 | 3 | 0 | 1 | 2 | 3 |
| 2. Relevant history: diagnosis and management to date | 0 | 1 | 2 | 3 | 0 | 1 | 2 | 3 |
| 3. Currently available management options within standard care | 0 | 1 | 2 | 3 | 0 | 1 | 2 | 3 |
| 4. Management options evaluated within study^2^ | 0 | 1 | 2 | 3 | 0 | 1 | 2 | 3 |
| 5. Clinical equipoise | 0 | 1 | 2 | 3 | 0 | 1 | 2 | 3 |
| 6. Study purpose or question | 0 | 1 | 2 | 3 | 0 | 1 | 2 | 3 |
| 7. Reason for randomisation | 0 | 1 | 2 | 3 | 0 | 1 | 2 | 3 |
| 8. Process of randomisation | 0 | 1 | 2 | 3 | 0 | 1 | 2 | 3 |
|  |  |  |  |  |  |  |  |  |
| **Total score** | /24 | | | | /24 | | | |
| **Comments arising during rating Section 2 i:** | | | | | | | | |

^1^ Scale evaluating recruiter information provision harmonised with scale evaluating participant understanding to allow rating of misleading information provision and evidence of misunderstanding respectively.

^2^ Word ‘trial’ was replaced with word ‘study’ in all parameters.

1. **Study treatments**

**Circle the most appropriate box for the information provided by the recruiter and the participant understanding for each item:**

| **Item** | | **Recruiter information** | | | | | | | | **Participant understanding** | | | | | | | |
| --- | --- | --- | --- | --- | --- | --- | --- | --- | --- | --- | --- | --- | --- | --- | --- | --- | --- |
|  | | Mis-  information | | Absent | | Minimal  information | | Adequate  information | | Mis-  understanding | | Absent | | Minimal understanding | | Adequate understanding | |
| 9. Study arm 1 processes | | 0 | | 1 | | 2 | | 3 | | 0 | | 1 | | 2 | | 3 | |
| 10. Study arm 1 costs or disadvantages | | 0 | | 1 | | 2 | | 3 | | 0 | | 1 | | 2 | | 3 | |
| 11. Study arm 1 benefits or advantages | | 0 | | 1 | | 2 | | 3 | | 0 | | 1 | | 2 | | 3 | |
| 12. Study arm 2 processes | | 0 | | 1 | | 2 | | 3 | | 0 | | 1 | | 2 | | 3 | |
| 13. Study arm 2 costs or disadvantages | | 0 | | 1 | | 2 | | 3 | | 0 | | 1 | | 2 | | 3 | |
| 14. Study arm 2 benefits or advantages | | 0 | | 1 | | 2 | | 3 | | 0 | | 1 | | 2 | | 3 | |
| 15. Study arm 3 processes | | 0 | | 1 | | 2 | | 3 | | 0 | | 1 | | 2 | | 3 | |
| 16. Study arm 3 costs or disadvantages | | 0 | | 1 | | 2 | | 3 | | 0 | | 1 | | 2 | | 3 | |
| 17. Study arm 3 benefits or advantages | | 0 | | 1 | | 2 | | 3 | | 0 | | 1 | | 2 | | 3 | |
|  | |  | |  | |  | |  | |  | |  | |  | |  | |
| **Total score** | | /18 or /27 | | | | | | | | /18 or /27 | | | | | | | |
| **Comments arising during rating Section 2 ii:** | | | | | | | | | | | | | | | | | |

1. **Study procedures**

**Circle the most appropriate box for the information provided by the recruiter and the participant understanding for each item:**

| **Item** | | **Recruiter information** | | | | | | | | **Participant understanding** | | | | | | | |
| --- | --- | --- | --- | --- | --- | --- | --- | --- | --- | --- | --- | --- | --- | --- | --- | --- | --- |
|  | | Mis-  information | | Absent | | Minimal  information | | Adequate  information | | Mis-  understanding | | Absent | | Minimal understanding | | Adequate understanding | |
| 18. Advantages or benefits of study participation | | 0 | | 1 | | 2 | | 3 | | 0 | | 1 | | 2 | | 3 | |
| 19. Costs / risks of study participation | | 0 | | 1 | | 2 | | 3 | | 0 | | 1 | | 2 | | 3 | |
| 20. Option to refuse participation | | 0 | | 1 | | 2 | | 3 | | 0 | | 1 | | 2 | | 3 | |
| 21. Option to withdraw from participation | | 0 | | 1 | | 2 | | 3 | | 0 | | 1 | | 2 | | 3 | |
| 22. Options for further consultation to support decision making | | 0 | | 1 | | 2 | | 3 | | 0 | | 1 | | 2 | | 3 | |
| 23. Outlines any conflict of interests for Recruiter^3^ | | 0 | | 1 | | 2 | | 3 | | 0 | | 1 | | 2 | | 3 | |
| 24. Outlines measures to protect confidentiality of participant data | | 0 | | 1 | | 2 | | 3 | | 0 | | 1 | | 2 | | 3 | |
| 25. Outlines what happens if things go wrong^3^ | | 0 | | 1 | | 2 | | 3 | | 0 | | 1 | | 2 | | 3 | |
|  | |  | |  | |  | |  | |  | |  | |  | |  | |
| **Total score** | | / 24 | | | | | | | | / 24 | | | | | | | |
| **Comments arising during rating Section 2 iii:** | | | | | | | | | | | | | | | | | |

^3^ Rewording in line with text conventionally used in PILs [10] as the optimum plain English version

| **Section 3: Global judgements**  Respond to the following questions as applied up to the point in the discussion when the participant decides whether to take part in the study or at the end of the appointment if no decision is reached.  **Circle your response and provide evidence below.** | | |
| --- | --- | --- |
| 1. **Does the recruiter consistently convey a position of equipoise? What evidence do you have to support your judgement?** | | |
| Yes | Insufficient evidence | No |
| Evidence: | | |
| 1. **Do you believe the patient is in equipoise? What evidence do you have to support your judgement?** | | |
| Yes | Insufficient evidence | No |
| Evidence: | | |
| 1. **Do you believe the patient accepts randomisation as a way to determine treatment? What evidence do you have to support your judgement?** | | |
| Yes | Insufficient evidence | No |
| Evidence: | | |
| 1. **Do you believe that the patient is sufficiently informed by the end of the consultation to make an informed decision? What evidence to you have to support your judgement?** | | |
| Yes | Insufficient evidence | No |
| Evidence: | | |

| **Section 4: Ethnographic commentary**  There are no constraints on what can be included here. Add observations about **any elements of recruiter or participant contributions that stand out in this consultation** with regard to **what** is discussed, **how** it is discussed and **how** it is understood.  Observations from this section will be used to develop the next version of the measure so please add comments on key issues that you feel need capturing and are not yet captured elsewhere. Shorthand notes are perfectly acceptable rather than written prose. It is intended that this section should be completed in around 10 minutes. |
| --- |
| **Comments:** |
