# Supplementary material for: Informed consent in randomised controlled trials: further development and evaluation of the participatory and informed consent (PIC) measure
Source: Trials. 2023 May 2;24:305. doi: 10.1186/s13063-023-07296-y (PMC10155434; doi:10.1186/s13063-023-07296-y)
Supplement: Supplementary file 4 — Additional file 4. PIC version 3 instructions for use. [file 13063_2023_7296_MOESM4_ESM.docx]

**Participatory and Informed Consent measure**

**PIC V3 Instructions FOR USE**

The Participatory and Informed Consent measure (Wade et al. 2017) is designed for application to the audio recording of randomised controlled trial (RCT) recruitment consultations to evaluate the quality of information provided by the recruiter and evidence of patient understanding for informed consent. The current version of the measure (DevPICv3) comprises four sections incorporating quantitative and qualitative assessment.

Prior to using the measure, **the template provided in Appendix 1 should be customised with study specific information** based on information in the protocol, participant information sheet and/or agreed with the trial management group. This is essential for rating the information provided by the recruiter in Section 2 of the DevPICv3.

The following information provides guidance on completing each section of the DevPICv3.

**Section 1: Descriptive information**

Section 1 i contains descriptive information about the RCT and recruitment consultation along with details of the rater applying the DevPICv3. Raters should complete this section prior to rating the audio-recording. Section 1 ii should be completed following the rating of Section 2.

**Section 2: Recruiter information and participant understanding**

Section 2 comprises three themes i) scene setting ii) study treatments and iii) study procedures, with either 22 or 25 items for two or three arm RCTs respectively (as detailed in Appendix 1).

Each item is rated on a scale of 0 to 3 for the information provided by the recruiter and evidence of participant understanding of the information. Ratings should reflect **all talk** within the consultation relevant to each item. Guidance on rating items is as follows:

|  | **Rating** | **Guidance** |
| --- | --- | --- |
| **Recruiter information** | 0 Mis-information | Recruiter provides **incorrect information** relating to the item which is **not clarified or resolved** by the end of the consultation. |
|  | 1 Absent | Recruiter **does not provide any information** relating to the item. |
|  | 2 Minimal information | Recruiter provides **some** relevant information relating to the item.  N.B. If **any incorrect information** is given and **not clarified or resolved**, rate as ‘0’ |
|  | 3 Adequate information | Recruiter provides **all** relevant information relating to the item. |
| **Participant understanding** | 0 Mis-understanding | Evidence that the participant **misunderstands the information** and this is **not clarified or resolved** by the end of the consultation. |
|  | 1 Absent | **No evidence** of understanding or misunderstanding the information. |
|  | 2 Minimal understanding | Participant provides a **minimal response** to the information i.e. ‘yes’, ‘ok’, right’, ‘I see’. |
|  | 3 Adequate understanding | Participant **demonstrates understanding** of the information i.e. asking relevant questions, re-phrasing or paraphrasing what has been discussed. |

Ratings for each item are combined to provide a score for each theme and an overall total score for recruiter information and participant understanding, with this information recorded in Section 1 ii.

**Section 3: Global judgements**

This section is based on the entire consultation and asks for the rater’s overall judgement across four areas after the detailed rating from Section 2 has been applied. Each judgement requires a rating of ‘yes’, ‘insufficient evidence’, or ‘no’ with space provided for evidence to support the assessment.

**Section 4: Ethnographic commentary**

This section provides space for an ethnographic commentary of what occurs within the consultation. There are no constraints on what can be included. Observations are invited about any elements of recruiter or patient contributions that stand out in terms of what is discussed, how it is discussed and how it is understood.

**Appendix 1: Study specific guidelines for rating recruiter information**

| **Study name:** | OPTiMISE | | | | |
| --- | --- | --- | --- | --- | --- |
| **Study arm 1:** | Standard care | **Study arm 2:** | Reduction of medication | **Study arm 3** *(If applicable)*: | N/A |

The information documented here is used to score the information provided by the recruiter in Section 2 for each of the 22 or 25 items (two or three arm RCTs). The table below provides generic definitions for each item, along with space for study specific definitions. The study specific information should be developed within the study team and documented in the table (in the blue rows) prior to applying the PIC. Relevant information should be available from the RCT protocol, participant information sheet and/or agreed with the RCT management group.

Ratings should reflect **all talk** within the consultation that is relevant to each item. A single segment of talk may be rated against more than one item. For a score of 3 (adequate information), the recruiter must give **clear information** on **all** points as detailed in the item definition.

| **ITEM** | | **DEFINITION** | |
| --- | --- | --- | --- |
| 1. | Purpose of consultation | - Mentions the **study by name** | |
|  |  |  | - OPTiMISE |
|  |  | - Clarifies that the consultation is an opportunity for the participant to make a **decision about taking part** in the study | |
| 2. | Relevant history: diagnosis and management to date | - Describes **diagnosis** | |
|  |  |  | - Patient previously diagnosed with high blood pressure |
|  |  | - Discusses **relevant previous or current medical care** with the participant | |
|  |  |  | - Patient taking two or more medications to control blood pressure (anti-hypertensive medication). - States that the patient’s blood pressure is currently controlled with these medications. |
| 3. | Currently available management options within standard care | - Describes **options available in standard care** (available outside of the study)   *i.e. currently someone in your position with diagnosis X, will be offered A, B, C…* | |
|  |  |  | - Blood pressure is controlled by anti-hypertensive medication. - Different types of medication are often given in combination. |
| 4. | Management options evaluated within study | - Describes **options evaluated within the study**   *i.e. participants in the trial will be offered A, B, C…* | |
|  |  |  | - One group of patients will stay on their current medication. - The other group of patients will have one of their anti-hypertensive medications stopped |

| **ITEM** | | | | **DEFINITION** | |
| --- | --- | --- | --- | --- | --- |
| 5. | Clinical equipoise | | | - Conveys **uncertainty** as to which treatment will produce a better outcome for any individual | |
|  |  |  |  | - Describes the **balance** between **risks and benefits** of the study arms   *i.e. ‘study arm’ may help with [X, Y, Z] but it may also result in [A,B,C]* | |
|  |  |  |  |  | - Many people take drugs to lower their blood pressure and reduce their risk of stroke and heart attacks. After many years of treatment, the benefits of taking these drugs may become outweighed by the risk of falls and other side effects. |
|  |  |  |  | - Describes that there is currently **no available evidence** to compare the treatments | |
| 6. | Study purpose or question | | | - Describes how the study will collect evidence as to whether **one of the treatments/options is better** than the other | |
|  |  |  |  |  | - Patients have been invited to take part if they are aged 80 years or older, take two or more anti-hypertensive medications, and have blood pressure considered to be in the normal range. - The study is trying to find out if it is safe to reduce the number of anti-hypertensive medications prescribed to these patients i.e. blood pressure remains controlled with less medication. |
| 7. | Reason for randomisation | | | - **‘N’ groups of people** are needed | |
|  |  |  |  |  | - Two groups of people |
|  |  |  |  | - Describes that the groups will be **the same except for which treatment they receive** - Explains that the treatment **outcomes** can then be **compared** across the groups **to see if there is any difference** | |
| 8. | Process of randomisation | | | - Describes **allocating** people to treatment groups by **chance** - Explains what **chance the participant has of receiving each treatment/option** | |
|  |  |  |  |  | - 50/50 or equal chance |
|  |  |  |  | - Describes that chance allocation **avoids any bias** in allocating people to groups | |
| 9. | Study arm 1 processes | |  | - Discusses **what happens in relation to visits, treatment, assessments etc.** for participants randomised to study arm 1 | |
|  | - Standard care | |  |  | **Visits**   - Two visits at the GP surgery: 1) a baseline appointment with a researcher on the day of consent, and 2) a follow-up appointment with a GP or the research team at 12 weeks. - Randomisation at the baseline appointment.   **Assessments**   - At each visit the patient will answer questions about themselves, complete questionnaires and have their height, weight and blood pressure measured.   **Follow-up**   - After the 12 week visit, long-term follow-up of mortality and hospital admissions will be undertaken via NHS Digital’s patient tracking service. This will not require the participant to do anything else. |
|  |  | |  |  |  |
| **ITEM** | | | | **DEFINITION** | |
| 10. | | Study arm 1 costs or disadvantages | | - Discusses the **risks, costs and/or disadvantages** of study arm 1 | |
|  |  |  |  |  | - No direct risks related to being allocated to standard care. - However, taking many drugs may increase the risk of falls and sometimes death in older patients, particularly in those suffering from lots of medical conditions. |
| 11. | | Study arm 1 benefits or advantages | | - Discusses the **benefits and/or advantages** of study arm 1 | |
|  |  |  |  |  | - No clear additional benefits of being allocated to standard care. - However, the patient will know that they have contributed to research which helps develop better ways to care for people as they get older. |
| 12. | | Study arm 2 processes |  | - Discusses **what happens in relation to visits, treatment, assessments etc.** for participants randomised to study arm 2 | |
|  |  | - Reduction of medication |  |  | **Visits**   - Three visits at the GP surgery: 1) a baseline appointment with a researcher on the day of consent, 2) a routine safety follow-up appointment with a GP or other appropriate delegated healthcare professional at 4 weeks, and 3) a follow-up appointment with a GP or the research team at 12 weeks. - Randomisation at the baseline appointment.   **Assessments**   - At each visit the patient will answer questions about themselves, complete questionnaires and have their height, weight and blood pressure measured. - At the 4 week safety visit, the patient may be invited for a further follow-up visit to recheck blood pressure and adjust medication (dose or type) if an adverse event occurs or if blood pressure is uncontrolled (i.e. sustained above 150 mmHg).   **Self-monitoring**   - Patients are given the opportunity to measure their blood pressure at home with a blood pressure monitor provided by the research team.   **Follow-up**   - After the 12 week visit, long-term follow-up of mortality and hospital admissions will be undertaken via NHS Digital’s patient tracking service. This will not require the participant to do anything else. |
|  |  |  |  |  |  |

| **ITEM** | | **DEFINITION** | |
| --- | --- | --- | --- |
| 13. | Study arm 2 costs or disadvantages | - Discusses the **risks, costs and/or disadvantages** of study arm 2 | |
|  |  |  | **Risks**   - Reducing medication could result in a rise in blood pressure, leading to an increased risk of heart attack or stroke.   **Minimising risks**   - All patients will have the opportunity to monitor their blood pressure at home and will have their blood pressure carefully monitored by a GP/other healthcare professional, so the likelihood of a heart attack or stroke is very low. - If blood pressure does increase significantly, the GP will take appropriate action, either adjusting or restoring medication. |
| 14. | Study arm 2 benefits or advantages | - Discusses the **benefits and/or advantages** of study arm 2 | |
|  |  |  | - Patients may be less likely to suffer side effects from medication such as falls or other side effects which could affect quality of life. |
| 18. | Advantages or benefits of study participation | - Discusses the **wider advantages and/or benefits** of participating in the study   *i.e. closer monitoring of health, extra visits, follow-up* | |
|  |  |  | - All patients will have their medication reviewed and their blood pressure checked on at least two separate occasions within the trial, which may inform discussions with the patients and potential adjustment of medication outside of the trial. - Patients will know that they have contributed to research which helps develop better ways to care for people as they get older. |
| 19. | Costs / risks of study participation | - Discusses the **wider costs and/or risks** of participating in the study   *i.e. extra visits, questionnaires, longer period in follow-up* | |
|  |  |  | - The trial requires patients to visit the GP surgery on at least two occasions for ‘longer than usual’ appointments. This may be inconvenient for some patients with other commitments. |
| 20. | Option to refuse participation | - Informs participant of the **option to decline** participation in the study | |
| 21. | Option to withdraw from participation | - Informs the participant of the **option to withdraw** from the study at any time - Explains that withdrawing from the study **will not affect care** received | |

| **ITEM** | | **DEFINITION** | |
| --- | --- | --- | --- |
| 22. | Options for further consultation to support decision making | - Informs the participant that **further consultations or opportunities for discussion** are possible to assist with decision making about participation - Explains **who** the participant could/would see for a further consultation/discussion | |
|  |  |  | - The patient can contact the trial team for further information or discussion about the trial - The patient can also book a further appointment with the recruiting GP at the practice |
|  |  | - Explains **when** the further consultation or discussion could/would take place | |
|  |  |  | - At a convenient time for the patient |
| 23. | Outlines any conflict of interest for recruiter | - Outlines any **benefits to the recruiter or the recruiting organisation** if the participant agrees to take part | |
|  |  |  | - N/A |
| 24. | Outlines measures to protect confidentiality of participant data | - Discusses **confidentiality** of participant data and the measures in place to keep it secure | |
|  |  |  | - States that all data will be stored securely and kept confidential. |
| 25. | Outlines what happens if anything goes wrong | - Outlines **what happens if something goes wrong** whilst the participant is taking part in the study | |
|  |  |  | - Contact the trial team. |
